# Identifying and Managing Hibernating Myocardium: What’s New and What Remains Unknown?

**DOI:** 10.1007/s11897-018-0396-6

**Published:** 2018-06-29

**Authors:** Matthew J. Ryan, Divaka Perera

**Affiliations:** 1 The Rayne Institute, St Thomas’ Hospital, 4th Floor Lambeth Wing, Westminster Bridge Road, London, SE1 7EH UK; 2 Cardiovascular Division, King’s College London, London, UK

**Keywords:** Hibernation, Viability, Revascularisation, Ischaemic heart failure, Ischaemic cardiomyopathy, HFrEF

## Abstract

**Purpose of Review:**

Hibernation is an important and reversible cause of myocardial dysfunction in ischaemic heart failure.

**Recent Findings:**

Hibernation is an adaptive process that promotes myocyte survival over maintaining contractile function. It is innate to mammalian physiology, sharing features with physiological hibernation in other species. Advanced imaging methods have reasonable accuracy in identifying hibernating myocardium. Novel superior hybrid methods may provide diagnostic potential. New evidence supports the role of surgical revascularisation in ischaemic heart failure, but the role of viability tests in planning such procedures remains unclear. Research to date has exclusively involved patients with ambulatory heart failure: Investigating the role of hibernation in ADHF is a key avenue for the future.

**Summary:**

Whilst our understanding of hibernation pathophysiology has improved dramatically, the clinical utility of identifying and targeting hibernation remains unclear.

## Introduction

**Fig. 1:**
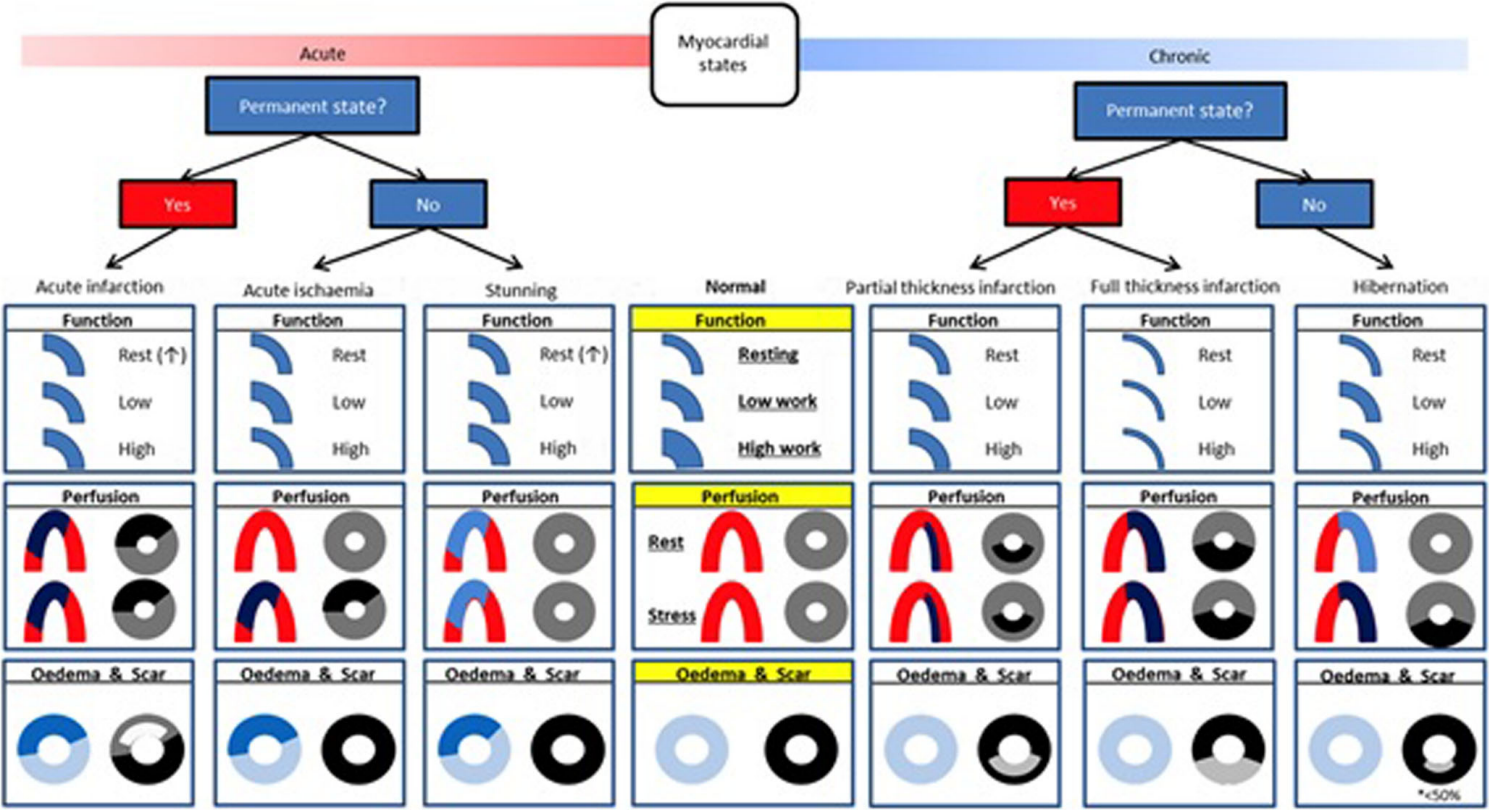
Taxonomy of myocardial segments in left ventricular systolic dysfunction. This should be viewed as an aid to classification rather than a decision tree. Function: thickness compared with ‘normal’ denotes resting state, with subsequent contractile reserve displayed with increase in segmental thickness. Stunned myocardium may display increase thickness at rest due to oedema though may not be readily appreciable. Perfusion: ‘horseshoe’ displays typical single photon emission computed tomography/positron emission tomography finding, whilst ‘donut’ displays cardiac magnetic resonance findings: single photon emission computed tomography/positron emission tomography: red = normal; pale blue = minimally decreased or normal; dark blue = decreased. Cardiac magnetic resonance: grey = normal; black = hypoperfusion/ischaemia. Oedema and scar: ‘donut’ displays typical T2-weighted (blue) and late gadolinium enhancement findings (black is normal, shades of grey represent late enhancement). (Reproduced with permission from McDiarmid et al. Taxonomy of segmental myocardial systolic dysfunction. European Heart Journal 2016: 38; 942–954)

Despite, and in part due to improving revascularisation techniques, ischaemic heart disease (IHD) remains the most prevalent cause of chronic heart failure in developed nations [1]. Even with modern drug and device therapy, 5-year mortality for ischaemic heart failure remains in excess of 50% [2•, 3]. Mortality events in ischaemic heart failure can be categorised as sudden deaths (predominantly due to arrhythmic and acute coronary occlusive events) or progressive pump failure leading to ADHF. IHD accounts for 40–60% of patients hospitalised with acute decompensated heart failure (AHDF) [4, 5] and ischaemic ADHF confers a worse in-hospital mortality than a non-ischaemic aetiology [6] (Fig. 1).

Studies across a range of modalities have demonstrated that most patients with ischaemic heart failure have a reduced left ventricular ejection fraction (HFrEF). Transmural infarction accounts for a significant proportion of this myocardial dysfunction. There was early recognition, however, that function is commonly impaired in myocardial segments that are free from ischaemic scar and have preserved metabolic activity, and also that a proportion of patients with coronary disease and HFrEF have no clinical or imaging evidence of prior infarction. Subsequent observations by Brundage and others suggested that contractile function in these regions commonly, but not inevitably, improved following revascularisation with coronary artery bypass grafting [7].

These observations led to Shahbudin Rahimtoola’s first description of myocardial hibernation [8]. This theory describes an adaptive downregulation of function in response to myocardial ischaemia, sacrificing contractile function to balance myocardial oxygen demand to the available supply and prevent myocyte necrosis. This process is an attractive pathophysiological target in decompensated heart failure; dysfunctional myocardium that could be “switched back on” to reverse progressive pump failure. Despite three decades of research, the role of hibernation in ischaemic HFrEF remains controversial. The last few years have, however, seen a steady stream of new data. This article will summarise and analyse recent progress in the identification and management of hibernation, later focussing on its role in ADHF.

### Recent Advances in Our Understanding of Hibernation

Early nuclear cardiology studies established that the degree of myocardial dysfunction in hibernation correlates with the extent of inducible perfusion defects [9]. Hibernation is therefore presumed to be an adaptation to chronic hypoperfusion, such that contractile function is chronically but reversibly downregulated [10•]. Despite ongoing research, the extent and chronicity of hypoperfusion needed to induce hibernation has been a contentious subject. Following brief periods of hypoperfusion and ischaemia contractile function is reduced. This process, termed “stunning”, has been observed experimentally [10•]. It may persist for some time, but a single episode of ischaemia normally results in a return to normal myocardial function over some hours once normal perfusion is restored. Rahimtoola’s initial description therefore hypothesised that the development of myocardial dysfunction at rest required the presence of sufficiently severe and permanent hypoperfusion for the myocardium to be subject to “chronic, painless ischaemia” [8].

In considering this theory, it is important to separate ischaemia (biochemically defined as state of insufficient oxygen delivery to match the myocardium’s metabolic requirement) from hypoperfusion (a relative reduction in perfusion that may or may not be adequate, depending on concurrent demand). Across the spectrum of normal physiological function, there is precise matching of coronary perfusion to myocardial oxygen demand, a relationship that is maintained in many pathological states but has been questioned in hibernation. Early animal work demonstrated biochemical ischaemia to be absent at rest, but controversy has remained with regard to perfusion. The ongoing controversy has centred on whether hibernating myocardium is constantly hypoperfused (and therefore supply and demand remain coupled) or whether hypoperfusion and ischaemia only occur in the context of increased physiological requirement (with supply and demand uncoupled and demand downregulated below the available supply at rest), e.g. on exertion.

Recent investigations in a well-validated swine model have shown that repeated ischaemia with normal perfusion in between episodes is sufficient to induce hibernation [11]. The severity of contractile dysfunction and remodelling increases in direct relation to reductions in coronary flow reserve. These observations have been confirmed in patients both in the ambulatory setting [12, 13] and through the induction of wall motion abnormalities via repetitive stress with treadmill exercise [14] and haemodialysis [15]. The key mechanism that induces hibernation therefore appears to be a reduction in coronary flow reserve [16], rather than reduced resting flow, an important observation when considering diagnostic tests for hibernation.

Early histopathological studies utilising ventricular biopsies taken at bypass surgery demonstrated consistent morphologic changes, characterised by myocyte de-differentiation (as opposed to degeneration) and varying degrees of loss of contractile apparatus (sarcomeres, sarcoplasmic reticulum and T-tubules) [17]. These changes are associated with abundant glycogen deposition, strands of rough endoplasmic reticulum and reductions in mitochondrial oxygen consumption despite preserved numbers. The cellular changes are perfusion dependent, being consistently more prevalent in the ischaemia-prone endocardium, compared to the epicardium, and directly correlated with the severity of stenosis within the subtending coronary artery [17]. There is a spectrum of change, from early to advanced, in different patients and in different vascular territories. There are inverse relationships between the extent of histological abnormality and the probability of improvement in contractile function following revascularisation [18]. These findings support the proposed model of hibernation as a chronic adaptive response that may eventually become so established it becomes irreversible.

Recent years have seen further progress in our understanding of the pathophysiology of hibernation. Research in swine has demonstrated the reversibility of histological changes and myocyte proliferation associated with functional recovery following revascularisation [19•]. Clearer evidence exists that the energetic state of hibernating myocardium is also preserved at rest [20, 21]. A fall in beta-adrenergic density likely modulates the reduced contractile reserve to limit myocardial oxygen demand to match perfusion reserve [22, 23]. Fascinatingly, the metabolic signature of hibernating hearts in patients appears comparable to those observed in bears during seasonal hibernation [24], where cardiac index falls to a mean of 0.63 l/min/kg [25]. This supports the presumption that hibernation is an innate adaptive process of mammalian myocardium that is activated to deleterious effect in ischaemic heart failure. Emerging evidence also confirms hibernation as a protective mechanism permitting greater tolerance of acute and/or persistent ischaemia. There is an associated activation of an endogenous gene program of cell survival [26], not observed in normal or infarcted myocardium. Experiments in small mammals (European ground squirrels and woodchucks) have demonstrated a marked degree of protection against ischaemia/reperfusion injury due to the induction of controlled improvements in mitochondrial energy and antioxidative capacity [27].

### Identifying Hibernation in Ischaemic HFrEF

Moving to the clinical aspects, the first challenge in managing hibernation is to accurately identify it. The definition of hibernation is retrospective: the observation of functional recovery following revascularisation. Contemporary tests attempt to prospectively predict this recovery, with variable success. The term “viability” is commonly used to prospectively classify of all dysfunctional but non-infarcted myocardium. The term is often, but incorrectly, used interchangeably with hibernation: whilst hibernation represents a substantial proportion of the dysfunction observed in patients with ischaemic heart failure, other pathophysiological processes may result in similar reductions of contractile function in non- infarcted segments. These include cardiomyopathy, acute ischaemia, transient myocardial stunning, adverse remodelling, and partial thickness infarction. As with hibernation, these states may co-exist within the ventricle or a single myocardial segment.

Early phenotyping refers to the use of cardiac biomarkers, imaging tests and physiological assessment to identify aetiology at the time of initial presentation with heart failure. The supposition is that by understanding the pathological mechanisms at work in any given patient, treatment can be personalised and tailored to improve outcome. Phenotyping ischaemic HFrEF is challenging: initially one must identify coronary disease, then subsequently determine causality (is this ischaemic heart failure or non-ischaemic heart failure with “bystander” coronary disease?) and subsequently sub-classify the pathological state of each region of myocardium to potentially guide therapy. It is the last aspect that is most relevant to hibernation. International guidelines support the use of imaging to identify the presence of IHD in acute heart failure [28] but the true clinical benefit of detailed phenotyping is unknown. Substantial progress has been made, however, in the available phenotyping modalities to address these issues. Dobutamine stress echocardiography (DSE), myocardial perfusion imaging (SPECT), late gadolinium enhanced cardiac MRI (LGE-CMR) and invasive coronary angiography are all in routine clinical use. A recent review article provides a succinct but highly relevant summary of the role of each modality in identifying myocardial pathology [10•].

#### Cardiac Magnetic Resonance Imaging

Of the imaging modalities available for phenotyping ischaemic heart failure, the most marked recent developments have been in cardiac MRI. IHD can be identified either through the demonstration of ischaemic patterns of myocardial scar or the presence of inducible perfusion defects using vasodilator stress testing. Acute infarction can be identified through areas of myocardial oedema, an assessment not available in any other imaging modality. Quantification of late gadolinium enhancement (LGE) provides useful prognostic information in patients with- and without- left ventricular dysfunction [29].

The extent of ischaemic-pattern left ventricular myocardial infarction determined by LGE-CMR has an excellent specificity for the diagnosis of ischaemic heart disease. The simultaneous acquisition of reference-standard data on ventricular systolic function further increases clinical and prognostic utility. Not all patients with ischaemic HFrEF, however, will have imaging evidence of prior infarction. This raises a concern if LGE-CMR is used in isolation: the sensitivity of LGE-CMR alone for an ischaemic aetiology may be reduced, in particular in patients with a greater volume of reversible hibernation, assuming matched levels of dysfunction. This could lead to missed opportunities if an incorrect diagnosis of dilated cardiomyopathy is made. The present role of CMR is, therefore, sub-classifying myocardial pathology and planning revascularisation once the diagnosis of ischaemic heart failure has been made.

As discussed above, cardiac MRI is unable to prospectively identify hibernation per se. Given, however, that hibernation accounts for the majority of dysfunctional but non-infarcted myocardium; its presence is (imperfectly) inferred from the absence of scar. The initial demonstration of MRI viability assessment using LGE was in 2000 [30], and since that time has become available in many centres. The technique utilises a morphological assessment, with scarred and non-scarred myocardium showing different signal intensities due to altered gadolinium kinetics. In clinical practice, a threshold of < 50% infarct transmurality is used to define viability, but in reality, there is a continuous and inverse correlation between % transmurality and the probability of recovery of contractile function following revascularisation [30]. The clinical threshold demonstrates high sensitivity but relatively poor specificity for functional recovery (80 and 62%, respectively) [31]. This can be improved through the addition of dobutamine stress, assessing contractile reserve within dysfunctional myocardium. Combining the two assessments results in the highest accuracy for any contemporary test of myocardial viability (sensitivity 80% and specificity 78%) [31, 32]. Vasodilator stress perfusion CMR is also highly accurate and reproducible in the assessment of inducible ischaemia due to coronary disease [33]. There is a lack of specific data on the diagnostic utility of combined LGE and perfusion imaging in this context and given that both ischaemia and viability are critical to the development of hibernation, combining the techniques may further improve predictive accuracy. This is a key avenue of future investigation.

#### Echocardiography

Echocardiography is less expensive and more widely available than CMR and is central to establishing the diagnosis of HFrEF. Features such as regional wall motion abnormalities, variation in wall thickness and ischaemic mitral regurgitation can point towards an ischaemic aetiology. Resting echocardiography has some utility at ruling in or ruling out viable myocardium at the extremes of the spectrum of segmental dysfunction. Preserved end diastolic wall thickness (EDWT) > 8 mm shows reasonable diagnostic accuracy for hibernation (sensitivity 75%, specificity 80%), whilst values < 6 mm nearly, excludes recovery (negative predictive value 93%) [34]. The presence of some contractility at rest (i.e. hypo- as opposed to a- or dys-kinesia) and normal myocardial reflectivity when compared to normally contracting segments also appear to have some diagnostic utility [35]. Whilst these levels of accuracy are insufficient to guide therapy alone, their utility in a hybrid diagnostic algorithm, where formal viability tests are reserved, for example, for a “grey-zone” of EDWT between 6 and 8 mm, warrants future investigation.

Stress echocardiography (DSE) remains the most widely available test of myocardial viability. Investigations of DSE have focussed on the minimum number of viable segments needed to predict a significant improvement in global LV function following (predominantly surgical) revascularisation. These have shown that demonstrating 4–6 viable segments has the best diagnostic performance, with a lower sensitivity but higher specificity than CMR (66 and 89%, respectively). A biphasic myocardial response, where contractility improves at low dose then deteriorates with increasing stimulation appears to have the greatest diagnostic accuracy [36]; the presumption here is that if both the absence of infarction (through initial contractile reserve) and the presence of inducible ischaemia (through subsequent deterioration) are proven, a complete substrate for recovery with revascularisation is identified. Early investigations of strain techniques demonstrate little incremental benefit [37], but their higher reproducibility may eliminate problematic issues with inter-observer variability and merit further study.

#### Cardiac Catheterisation

Cardiac catheterisation in the form of invasive coronary angiography is a frequently used diagnostic test in suspected hibernation, particularly in decompensated patients in whom a coexisting acute coronary syndrome is considered. Angiography permits detailed assessment of the coronary vasculature, as well as the acquisition of other useful prognostic data, such as left ventricular end diastolic pressure (LVEDP). Procedural duration and the deleterious effects of atrial fibrillation on diagnostic accuracy are significantly reduced compared with other modalities. Avoiding pharmacological stress may benefit patients who are acutely haemodynamically compromised. Contrast nephropathy remains a risk, given the frequency of acute or chronic renal impairment.

Whilst ideal for detecting coronary disease, used in isolation invasive angiography cannot determine causality. As discussed above, the extent of angiographic disease or myocardial jeopardy required to induce hibernation has not been determined, though a continuous relationship between coronary disease burden and likelihood of an ischaemic aetiology can be hypothesised. Angiographic stenosis severity also correlates poorly with objective perfusion assessments, a factor likely to be as important in ischaemic heart failure as it is in chronic stable angina [38]. Finally, invasive angiography is currently unable to make any assessment of the condition of the myocardium, requiring further imaging studies to be undertaken and considered against the results of the angiogram.

Detailed physiological tests, such as fractional flow reserve (FFR) or instantaneous wave-free ration (iFR) might provide relevant data on physiological stenosis severity. These tests have not been validated in HFrEF; however, the diagnostic thresholds required to induce hibernation are likely to be substantially lower than those used to predict ischaemia in those with normal ventricular function (FFR < 0.80 and iFR < 0.89, respectively). Novel physiological markers, such as the index of microcirculatory resistance (IMR) and wave intensity analysis (WIA) offer the opportunity to simultaneously assess myocardial physiology, but at present are restricted to research environments [39•, 40]. Very early data suggest invasive electro-anatomic indices, such as voltage mapping, also merit exploration [41, 42].

#### Novel Imaging Modalities

Novel imaging modalities under development may permit more accurate characterisation of hibernating myocardium. It is notable that, with the exception of high-dose DSE, each of the testing modalities currently available assesses a single aspect of the physiology of hibernation. Advanced intracoronary physiology, hybrid PET/MRI, PET/CT and stress/LGE-CMR all benefit from facilitating simultaneous assessment of multiple facets of hibernation physiology. The integration and co-registration of metabolic, perfusion, ischaemia and morphologic imaging with the distribution of the coronary vasculature could well unlock the accurate prospective identification of hibernating myocardium.

### Revascularisation in the Management of Ischaemic HFrEF

The role of revascularisation in ischaemic HFrEF has long been contentious, even without consideration of hibernation. After many years and conflicting data on the role of coronary artery bypass grafting (CABG) in ischaemic HFrEF, 10-year follow-up of the STICH trial recently confirmed a statistically significant benefit in cardiovascular mortality for CABG plus medical therapy over medical therapy alone [43••]. This randomised controlled trial enrolled 1212 patients with ischaemic HFrEF between 2002 and 2007. Randomisation was 1:1, with participating centres in 26 countries. All patients had an LV ejection fraction < 35% and severe coronary disease amenable to CABG. There was a substantial rate of procedural complications, with 10% of patients having died or remaining in hospital after 30 days. As a result, the overall benefit was modest: no significant reduction in mortality was observed at 5 years [44] and an 8% absolute reduction could only be demonstrated after 10 years of follow-up to develop [43••]. The reduction in a combined endpoint of death or rehospitalisation was more significant. As a result of the STICH trial data, revascularisation is now recommended as a treatment for patients with ambulatory heart failure and ischaemic left ventricular dysfunction in American [45] and European [46] guidelines.

Whilst percutaneous coronary intervention (PCI) is the most frequently used treatment for an acute coronary syndrome, there has been no prospective randomised investigation of the utility of angioplasty in the management of stable ischaemic HFrEF. Observational data demonstrates that treatment with PCI can result in comparable improvements in ventricular function, with some evidence that revascularisation of chronic total occlusions (CTO) provides particular benefit [47•]. There are theoretical advantages (fewer early adverse events and the ability to tailor revascularisation to the distribution of viable myocardium) and disadvantages (difficulty achieving complete revascularisation and risk of stent thrombosis), but no reliable data exist to support these. In the absence of specific evidence, current guidelines reserve angioplasty for those not suitable for treatment with CABG.

The REVIVED-BCIS2 trial is a multicentre UK study led by our group and will be the first randomised evaluation of the impact of revascularisation via PCI on mortality, morbidity and left ventricular function in patients with severe ischaemic HFrEF [48]. With 400 (of 700) patients enrolled to date, this is already the largest randomised assessment of percutaneous revascularisation in ischaemic left ventricular dysfunction. This carefully characterised cohort will provide unique opportunities to study a number of aspects of revascularisation in ischaemic left ventricular dysfunction. Some aspects, such as effects on hospitalisation and ventricular function, will be highly relevant to those interested in managing ischaemic heart failure.

#### Should Hibernation Be Considered in Revascularisation?

Whilst emerging evidence supports the role of revascularisation in ischaemic HFrEF, specific data for the role of hibernation/viability testing in determining revascularisation outcomes remain limited. There is clear evidence of greater improvements in function with an increasing burden of hibernation [49]. The idea of personalising revascularisation through the use of viability testing is attractive; it is apparent that many patients derive no benefit from either CABG or PCI procedures despite being exposed to all of the morbidity and mortality risks associated with them. Limiting exposure to those with the potential to benefit would be expected to improve overall outcomes and resource utilisation. These benefits, however, would only be applicable if viability testing is indeed a relevant marker of prognostic benefit.

Although often used as a surrogate endpoint, there is sparse data linking viability testing and myocardial functional recovery to improvements in hard outcomes (i.e. mortality and heart failure hospitalisation), with conflicting results [50, 51]. This is particularly relevant when considering the dichotomous classification of viability using current tests; a minimum thickness of viable myocardium is required to observe functional recovery or contractile reserve, an observation that may convince the clinician to defer revascularisation where the viable rim is “insignificant”. Hibernation has, however, been established as a potential substrate for ventricular tachyarrhythmias that may contribute to sudden arrhythmic death [52•]. Such outcomes, which revascularisation might mitigate [53], could be driven by regions with small volumes of residual viable tissue insufficient for contractile recovery but substantial enough to predispose to arrhythmias. On this basis, we should be hesitant about extrapolating functional recovery data into outcome benefits, and prospectively investigate the role of viability imaging in improving revascularisation outcomes.

Soon after the initial case reports, associations were made between the provision of revascularisation in those with significant viable myocardium and improved mortality in ischaemic heart failure. Allman and colleagues [54] meta-analysed observational studies assessing the interaction between viability and outcomes in patients undergoing revascularisation in ischaemic HFrEF. Including data from 3088 patients with a mean LV ejection fraction of 32%, the analysis demonstrated significant differences in outcome between patients with significant dysfunctional but viable myocardium dependent on revascularisation status (annual mortality 15% in patients who were medically managed vs. 3.2% in those who underwent revascularisation). This effect was not observed in those without residual viability (7.7 vs. 6.2%, respectively). There was clear recognition, however, as to the potential bias in this data. Older patients, those with more complex coronary disease and those with more impaired ventricular function might be exposed to selection bias and confound the results. Further prospective study was required.

There were several subsequent attempts to address the question of viability in guiding revascularisation, which struggled with low recruitment despite the best efforts of the investigators [55, 56]. The next substantial data on the subject came again from the STICH trial, although the trial protocol held no requirement for the assessment of viability, eligibility was determined on the basis of complexity of coronary disease and LV ejection fraction alone. A viability sub-study from the initial phase of the trial was published in 2012 [57]. The results indicated no significant difference in mortality outcomes between those who did and did not have significant residual viability, once adjusted for other baseline variables. The data must, however, be interpreted with significant caution; the sub-study was non-randomised, retrospective and viability was assessed dichotomously on the basis of greater or less than five viable myocardial segments. This led to the notable difference in mortality on univariate analysis, suggesting the baseline cohorts were not equivalent. In addition, data contained in the supplementary appendices shows the significant benefit of CABG on the combined endpoint of death and rehospitalisation is observed only in the viable cohort, including on multivariate analysis.

In contrast to the STICH trial, recruitment to the REVIVED-BCIS2 trial is dependent on presence of a significant degree of presumed myocardial hibernation (viability in at least four myocardial segments amenable to revascularisation). The eligibility requirements will also make this the first adequately powered prospective study of viability-guided management. Pre-planned sub-analyses of the data will investigate the role of hibernation in determining outcomes in both the revascularised and medically managed arms of the trial.

### Hibernation in Acute Decompensated Heart Failure

Whilst hibernating myocardium may remain stable over months [58], the preservation of myocyte integrity remains critically balanced and there is a risk of necrosis following a further acute physiological insult [6] or if left untreated for a prolonged time [59]. The risk of necrotic transformation is likely to be particularly high where resting flow and/or coronary flow reserve are markedly reduced. Acutely decompensated heart failure represents such an insult, with both LV end diastolic pressure and systemic hypotension resulting in further deteriorations in perfusion [6, 60]. Additionally, ADHF may be triggered by the new development of hibernation in those who have presented without an ACS. Given the high mortality of ischaemic ADHF and the greater relevance of contractile function compared to arrhythmic risk, the issues of hibernation are particularly relevant. Despite this, almost all hibernation research to date has involved participants with ambulatory heart failure. In all of the evidence base discussed above, an unstable condition was a specific exclusion criterion to trial recruitment. At present, the management of hibernation in ADHF must therefore be cautiously extrapolated from the stable setting.

Whilst not specifically investigated, identifying hibernation in ADHF is likely to be more challenging. Whilst resting transthoracic echocardiography remains the mainstay of diagnosing LVSD, dobutamine-stress testing is inadvisable in decompensated heart failure. Coronary angiography remains the diagnostic investigation of choice, particularly in patients where acute coronary syndromes cannot be excluded on clinical grounds alone.

CMR has received considerable attention for phenotyping in acutely presenting cardiology patients, and there is no major reason to believe LGE-CMR assessment would be invalidated by decompensation. That said, dysfunction due to adverse loading conditions may mimic hibernation, potentially leading to reduced specificity. Vasodilator stress perfusion may also be affected by the adverse loading conditions and venous pressure in decompensated heart failure, factors which have not been specifically investigated. The MRI environment and long-scan time may also be unsuitable for unstable patients. Differentiating myocardial oedema and established scar is challenging, particularly relevant to patients presenting with ischaemic ADHF, where determining whether an acute coronary event has occurred is often challenging and confounded by a prior history of IHD and modest elevations in cardiac biomarkers. Despite the majority of patients presenting without clinical evidence of ACS, troponin elevation is near ubiquitous in patients hospitalised with ischaemic ADHF, being observed in 85% of patients [61].

Such troponin elevations have recently been shown to be highly correlated with the extent of hibernation but not with established scar, suggesting subacute injury in hibernating myocardium as the mechanism of troponin release [62•]. The use of inotrope therapy is likely to pose particular risk in such regions, underlying their adverse effect on outcome despite initial symptomatic benefit, when compared to vasodilator and diuretic therapy that reduce LVEDP and myocardial oxygen demand. A significant proportion of patients develop elevated troponin levels within 3 days of admission despite normal baseline levels, suggesting a progressive injury to hibernating tissues that should encourage the identification of strategies for myocardial protection in the acute setting, particularly given that troponin elevation is a powerful predictor of mortality [63]. Given the previous data suggesting poor outcomes with their use, this makes a stronger case for the avoidance of inotropes, where substantial degrees of hibernation are suspected. Further strategies might include tailored pharmacotherapy (GLP-1 antagonists are under investigation [64]), mechanical circulatory support and acute revascularisation.

Observational evidence for the role of acute revascularisation in decompensated HFrEF is extremely limited, in part because it is rarely utilised in current clinical practice. Angiography remains underutilised in the acute setting, with recent data from the BIOSTAT-CHF programme demonstrating that only 12.5% of patients underwent angiography within 30 days of decompensation [65•]. Of 170,000 patients across three large ADHF registries, only 2–4% received inpatient revascularisation [6]. There is a small amount of retrospective data to suggest improved outcomes in ADHF patients who have undergone prior revascularisation, data that will again be subject to significant selection bias. As the benefit of revascularisation in the management of acutely decompensated heart failure is not defined, clinicians may take a pragmatic approach. In our centre, we have a low threshold for coronary angiography performed during the index admission. This permits early risk stratification and ongoing planning. Inpatient revascularisation is performed only where there is concern for developing cardiogenic shock or an inotrope requirement, or an ACS cannot be excluded on clinical grounds (including TIMI flow grade < 3). In patients without these features, revascularisation is initially deferred. Subsequent management strategy is discussed at a multidisciplinary forum and with the patient, with consideration given to enrolment in relevant clinical trials. Future investigations of the role of acute revascularisation in ADHF would be welcomed.

## Conclusions

The last decade has seen significant advances in our understanding of hibernation and its role in ischaemic left ventricular dysfunction. In particular, progress has been made in pathophysiological research, relevant diagnostics (particularly coronary physiology and CMR) and revascularisation techniques (including reducing surgical risk, complex/CTO angioplasty and mechanical circulatory support). These have contributed to far greater understanding of the role of revascularisation in ischaemic heart failure. Our knowledge of the utility of these techniques in the setting of decompensated heart failure remains primitive and is an essential avenue for future investigation.
